# Machine learning methods for metabolic pathway prediction

**DOI:** 10.1186/1471-2105-11-15

**Published:** 2010-01-08

**Authors:** Joseph M Dale, Liviu Popescu, Peter D Karp

**Affiliations:** 1 Bioinformatics Research Group, SRI International, 333 Ravenswood Ave, Menlo Park, CA, 94025, USA

## Abstract

**Background:**

A key challenge in systems biology is the reconstruction of an organism's metabolic network from its genome sequence. One strategy for addressing this problem is to predict which metabolic pathways, from a reference database of known pathways, are present in the organism, based on the annotated genome of the organism.

**Results:**

To quantitatively validate methods for pathway prediction, we developed a large "gold standard" dataset of 5,610 pathway instances known to be present or absent in curated metabolic pathway databases for six organisms. We defined a collection of 123 pathway features, whose information content we evaluated with respect to the gold standard. Feature data were used as input to an extensive collection of machine learning (ML) methods, including naïve Bayes, decision trees, and logistic regression, together with feature selection and ensemble methods. We compared the ML methods to the previous PathoLogic algorithm for pathway prediction using the gold standard dataset. We found that ML-based prediction methods can match the performance of the PathoLogic algorithm. PathoLogic achieved an accuracy of 91% and an F-measure of 0.786. The ML-based prediction methods achieved accuracy as high as 91.2% and F-measure as high as 0.787. The ML-based methods output a probability for each predicted pathway, whereas PathoLogic does not, which provides more information to the user and facilitates filtering of predicted pathways.

**Conclusions:**

ML methods for pathway prediction perform as well as existing methods, and have qualitative advantages in terms of extensibility, tunability, and explainability. More advanced prediction methods and/or more sophisticated input features may improve the performance of ML methods. However, pathway prediction performance appears to be limited largely by the ability to correctly match enzymes to the reactions they catalyze based on genome annotations.

## Background

A key step toward understanding an organism's metabolism is the construction of a comprehensive model of the network of metabolic reactions taking place in the organism. Although a number of such models have been constructed through painstaking literature-based manual curation [1,2], such an approach obviously cannot scale to hundreds of sequenced genomes. Therefore, methods are needed for computational characterization of metabolic networks.

That task can involve two subtasks. (1) The *reactome prediction *problem: Given the annotated genome for an organism, predict the set of metabolic reactions catalyzed by the organism; that is, predict associations between enzymes and the reactions they catalyze. (2) The *pathway prediction *problem: Given the reactome of an organism and its annotated genome, predict the set of metabolic pathways present in the organism. In the current work we take the reactome as predetermined by other methods, and focus on developing improved pathway prediction methods. Pathway prediction can involve predicting pathways that were previously known in other organisms, or predicting novel pathways that have not been previously observed (*pathway discovery*). Our methodology does the former, predicting pathways from a curated reference database.

We have previously developed a method, called PathoLogic [3], for automatically constructing a Pathway/Genome Database (PGDB) describing the metabolic network of an organism, meaning the metabolic reactions catalyzed by enzymes in the organism and their organization into pathways. Based on the assumption that experimentally defined metabolic pathways are conserved between organisms, PathoLogic uses the MetaCyc [4] reference pathway database as a template for predicting the metabolic pathways of a newly sequenced organism. MetaCyc version 13.5 contains 1,400 experimentally characterized pathways curated from the literature for all domains of life.

Prediction of metabolic pathways in addition to the reaction network is important because pathways provide a higher level of organization that facilitates human comprehension of the network, and pathways suggest reactions and enzymes that may be initially missing from the model because of omissions in the genome annotation [5]. Prediction of pathways is hard for three reasons. (1) Errors and omissions in genome annotations introduce noise into the primary source of evidence for pathways, namely, the set of metabolic enzymes in the genome. (2) Enzymes that catalyze "promiscuous" reactions -- reactions that participate in multiple pathways -- are ambiguous in supporting the presence of more than one pathway. In the version of MetaCyc used for this work, 4,558 reactions participate in pathways. Of these, 779 reactions (17%) appear in at least two pathways. (3) Groups of variant pathways in MetaCyc (pathways that carry out the same biological function) often share several reactions, making it difficult to distinguish which variant is present.

At the core of PathoLogic is an algorithm for predicting which subset of pathways from MetaCyc is present in the organism. The algorithm incorporates rules and heuristics developed and tuned over several years, using feedback from biologists to improve the accuracy of the predictions.

However, the PathoLogic algorithm suffers from several limitations, which we aim to address in this work. (1) As MetaCyc grew in size, PathoLogic began to make more false positive pathway predictions. (2) The logic of the PathoLogic algorithm is hard-coded, with complicated interactions between various rules, making the algorithm difficult to maintain and extend. (3) PathoLogic provides limited explanations to the user as to why a particular pathway was included in, or excluded from, its predictions. Better quality explanations are needed. (4) The algorithm is limited to Boolean predictions and only a coarse measure of prediction confidence is provided by the number of reactions in the pathway whose enzymes are known in the organism. Thus, it is difficult to filter the predictions with a probability cutoff.

Our goal, therefore, is to develop a pathway prediction algorithm that is *data-driven*, *transparent*, and *tunable*. By *data-driven *we mean that the parameters determining the interaction of various predictive features are separate from the detailed logic of the predictor, and can be trained and modified at will. By *transparent *we mean that the evidence for or against a given pathway's presence can be presented to the user. By *tunable*, we mean that the algorithm provides a fine-grained measure of prediction confidence, such that by varying a threshold at which a prediction is made, the user can trade off sensitivity for specificity. To satisfy these goals, we have carried out the following work. (1) To quantify the performance of our new prediction algorithms, and compare them to the existing methods, we constructed a large pathway "gold standard" containing data on the presence or absence of metabolic pathways in various organisms. (2) We designed and implemented 123 features for evaluation in the new prediction algorithms. This set includes features used by PathoLogic, but consists mostly of new features. (3) We applied several commonly used machine learning (ML) algorithms to the pathway prediction problem. The best resulting ML-based algorithm achieved a small improvement over the performance of PathoLogic.

## Methods

**Figure 1:**
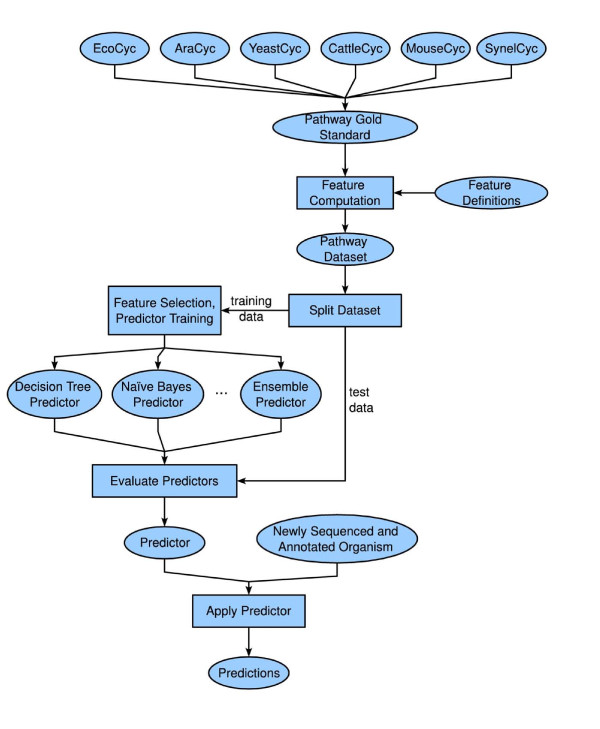
**Procedure for applying machine learning methods to metabolic pathway prediction**. Data from curated pathway/genome databases (PGDBs) are gathered into a "gold standard" collection. Features are defined using biological knowledge, and their values are computed for all pathways in the gold standard. The resulting dataset is split into training and test sets. Training data are used to perform feature selection and parameter estimation for multiple predictor types. Test data are used to evaluate the predictors. The predictor which performs best on the test set will be applied to data from newly sequenced and annotated genomes to perform metabolic network reconstruction.

Figure 1 provides an overview of the process of applying ML methods to pathway prediction. We describe each step in detail below.

### Construction of a Gold Standard Pathway Collection

To train our machine learning algorithms and validate them against existing methods (namely, PathoLogic), we constructed a "gold standard" dataset containing known information about which pathways are present and absent in a variety of organisms. The full gold standard dataset can be found in Additional file 1; here we describe the content and construction of the dataset.

The gold standard dataset currently contains 5,610 entries that describe pathway presence and absence in six organisms: *Escherichia coli *K-12 MG1655, *Arabidopsis thaliana *columbia, *Saccharomyces cerevisiae *S288c, *Mus musculus*, *Bos taurus*, and *Synechococcus elongatus *PCC 7942. The data are derived mainly from the corresponding PGDBs for these organisms: EcoCyc [1] version 13.0, AraCyc [6,7] version 4.5, YeastCyc [8] version March 2009, MouseCyc [9] version 1.41, CattleCyc [10] version 1.2, and SynelCyc version 1.13.5 (unpublished), all of which have undergone significant manual curation. Data from EcoCyc are entirely derived from manual literature curation. AraCyc and YeastCyc were built using an older version of PathoLogic, but have undergone extensive manual curation to remove false positive pathway predictions and to introduce additional pathways from the literature that were not present in MetaCyc. MouseCyc, CattleCyc, and SynelCyc were built within the past 1 to 2 years using PathoLogic and have undergone subsequent manual curation: MouseCyc by curators at the Jackson Laboratory, CattleCyc at the University of Illinois, and SynelCyc in our group at SRI International.

Each element of the gold standard dataset is a triple of the form *(organism, pathway, is-present?)*, asserting that a *pathway *(referring to a pathway object in MetaCyc) is present in or absent from an *organism*, depending on whether *is-present? *is "true" or "false". Table 1 shows the number of positive and negative instances for each organism in the gold standard.

**Table 1:** Number of positive and negative pathways for each organism in the gold standard dataset

organism	positives	negatives	total
*Escherichia coli *K-12 MG1655	235	1035	1270
*Arabidopsis thaliana *columbia	297	971	1268
*Saccharomyces cerevisiae *S288c	119	777	896
*Synechococcus elongatus *PCC 7942	171	778	949
*Mus musculus*	203	754	957
*Bos Taurus*	151	119	270

As noted, different methods were used to construct the PGDBs for the organisms represented in the gold standard, and these have undergone differing amounts of curation. For this reason, different rules were used to select examples from each organism to include in the gold standard. Two rules, however, applied to all organisms. First, we added as positive examples in the gold standard for each organism *O *all pathways that have been curated in MetaCyc as being present in *O*. That is, MetaCyc explicitly records the organism(s) in which each pathway was experimentally studied. Second, we added as gold standard negative examples for *O *all pathways *P *in MetaCyc such that none of the reactions of *P *had identifiable enzymes in the most recent genome annotation for *O*.

For *E. coli *we added as positives all pathways present in EcoCyc. We added as negatives all pathways not annotated in MetaCyc to be present in any strain of *Escherichia coli*. A similar approach was used for *A. thaliana*. We added as positives all pathways in AraCyc with noncomputational evidence (meaning the pathways were supported by an experimental evidence code, or by the evidence code "inferred by curator"). All pathways not present in AraCyc and not annotated in MetaCyc to any subspecies of *A. thaliana *were added to the negative set. Both EcoCyc and AraCyc have undergone extensive manual curation, and data is frequently propagated and synchronized between these databases and MetaCyc. We therefore expect these sets of examples to be relatively complete and robust.

YeastCyc is also extensively curated, but since its curation is not closely synchronized with curation of MetaCyc, we have been more cautious in adding yeast pathways to the gold standard dataset. The main source of positive examples for yeast is MetaCyc, which includes approximately 100 curated yeast pathways. In addition, we included all pathways from YeastCyc that were reviewed by a YeastCyc curator and are present in MetaCyc. A number of pathways in YeastCyc are not included in MetaCyc. Work is in progress on synchronizing YeastCyc and MetaCyc, and these pathways will be added to the gold standard as they are imported into MetaCyc. As negative examples, we included a set of pathways reported by the YeastCyc curators to have been deleted from YeastCyc. We also obtained an earlier version of YeastCyc, and included as negative examples pathways in the older version of YeastCyc that are no longer present in YeastCyc but still exist in MetaCyc (but are not annotated as occurring in yeast).

For MouseCyc, CattleCyc, and SynelCyc, all pathways present were added to the gold standard as positive examples. Pathways reported by the MouseCyc and CattleCyc curators to have been deleted were added as negatives, along with pathways recorded as deleted by the (relatively new) internal database logging mechanisms of Pathway Tools. The same was done for SynelCyc, although we were able to more carefully track pathway deletions because of our oversight of this process.

### Evidence Gathering and the PathoLogic Algorithm

PathoLogic is the state-of-the-art pathway prediction algorithm included in the Pathway Tools software suite [3,11]. Broadly, this program accepts as input the annotated genome of an organism, and outputs a database containing objects representing the genes, proteins, metabolites, reactions, and pathways of an organism. The batch mode of PathoLogic is used to construct the BioCyc database collection [4], currently containing 507 PGDBs.

An important step in metabolic network reconstruction is reactome inference: deriving associations between the proteins encoded by the genome and the reactions catalyzed by those proteins. These associations are the main source of evidence for the pathway inferences of both PathoLogic and our newer machine learning-based algorithms. As such, the quality of the inferred metabolic network is highly dependent on the completeness and correctness of the genome annotation and on the reaction associations. Linking of proteins with reactions is performed by the "enzyme matching" component of PathoLogic, which uses Enzyme Commission (EC) numbers, Gene Ontology (GO) annotations, and protein function or product names to link proteins in an annotated genome with MetaCyc reactions.

The PathoLogic algorithm selects as candidates for pathway prediction all pathways in MetaCyc that contain at least one reaction catalyzed by an enzyme in the target organism. The algorithm then iterates through the list of candidates, using a collection of manually derived rules [3] to decide whether or not to include a pathway in the new PGDB (that is, to predict the pathway as present in the organism). At each iteration the evidence for or against each remaining candidate pathway is recomputed based on the other remaining pathways. (This approach can affect rules comparing the evidence for multiple pathways, such as two variants of a biosynthetic function; only the variants not yet pruned are considered.)

The algorithm terminates when no more candidate pathways can be kept or pruned by the rules. If any undecided candidate pathways remain, PathoLogic errs on the side of inclusiveness, keeping all the candidates in the database. In the early development of PathoLogic, it was expected that most new PGDBs would be extensively reviewed by human curators, for whom it would be easier to remove false positives from the new PGDB than to comprehensively identify false negatives in MetaCyc. Although this is still a common use case, a primary motivation for the current work is to develop an algorithm in which the tradeoff between false positives and false negatives can be tuned for either meticulous individual database curation, or for high-throughput PGDB construction.

### Feature Extraction and Processing

On the basis of our past experience with the pathway prediction problem, we enumerated during this project a set of 123 alternative features that we thought might be relevant to this problem. The PathoLogic algorithm defines a set of 14 basic features which are combined in its rules for keeping or deleting pathways. In our research we used existing PathoLogic features, implemented new variations of PathoLogic features, and defined many novel features not used by the existing PathoLogic algorithm. Many of the features are quite similar to one another. The features include both categorical (Boolean or multivalued) features and numeric features. More fully, a feature can be a function of the pathway as well as the organism in which the pathway's presence is being predicted (the *target *organism). Some features do not depend on evidence in the target organism, but are only properties of pathways in the reference database, MetaCyc. (An example is the biosynthesis-pathway feature, which indicates whether a pathway is classified in MetaCyc as carrying out the biosynthesis of some compound.) A full description of all features considered can be found in Section 1 of Additional file 2. We expect that many of the feature names will be self-explanatory, but for concreteness we describe a subset of the features used in more detail below. Many features test whether reactions in pathways are *present*; this is true if the reaction is linked to an enzyme in the target database (DB), or if the reaction is annotated in the reference DB as occurring spontaneously. A *unique reaction *occurs in only one pathway. A *unique enzyme *catalyzes reactions in only one pathway. Reaction uniqueness is computed by PathoLogic with respect to the current set of candidate pathways, which changes throughout the algorithm. (An enzyme can be nonunique if it either catalyzes a single reaction that occurs in multiple pathways, or catalyzes multiple reactions that occur in different pathways. An enzyme catalyzing multiple reactions all occurring only in a single pathway is still unique to that pathway.) Since our algorithms do not currently have the notion of a changing candidate set, uniqueness is computed with respect to all MetaCyc pathways.

Many of the 123 features can be grouped into the following broad categories:

• **Reaction evidence**. Features based on the identification in the genome annotation of enzymes catalyzing reactions.

• **Pathway holes**. Features based on the pattern of holes (reactions missing enzymes) in a pathway. Is the pathway split by holes into several fragments? Does the pathway have long runs of holes? Does a biosynthetic pathway end with a hole, or a degradation pathway begin with one?

• **Pathway connectivity**. Features based on the context of the metabolic network (e.g., how well a pathway is connected to the rest of the metabolic network).

• **Genome context**. Features based on the position of the genes involved in each pathway in the target genome. For example, are there two reactions in the pathway whose enzymes are encoded by genes that are adjacent on the genome?

• **Pathway variants**. Features comparing the evidence for a pathway to alternative pathways (if any) that accomplish roughly the same biological function. For example, are the enzymes present in a pathway *V*_1 _a subset of the enzymes for a variant *V*_2_?

• **Taxonomic range**. Features based on the curated taxonomic range of the pathway. The taxonomic range is defined as the set of organisms in which the pathway is most likely to be present, and is specified by the MetaCyc curators.

• **PathoLogic-derived features**. Features used by PathoLogic but not classified in any of the previous categories.

• **Pathway properties**. Features computed based on pathway properties such as the number of reactions, and type of pathway (biosynthesis, degradation/detoxification, energy production).

Example features include the following:

• **some-initial-reactions-present**: This feature is true if some initial reaction of the pathway - a reaction with no preceding reactions in the pathway - is present.

• **taxonomic-range-includes-target**: Indicates whether the expected taxonomic range of the pathway (annotated by curators in MetaCyc) includes the target organism; false if the taxonomic range is not annotated, or if it does not include the target organism.

• **best-fraction-reactions-present-in-linear-path**: Computes the fraction of reactions present along each path from an input compound to an output compound in the pathway; returns the maximum such value. If the pathway is linear, this is equivalent to fraction-reactions-present.

• **evidence-info-content-norm-all**: Some reactions in metabolic networks participate in several pathways while other reactions belong to a single pathway. The more pathways a reaction participates in, the weaker is the evidence that an enzyme for that reaction provides for the presence of any one of those pathways. Thus, we defined several "information content"-based features, as exemplified here.

Let *m *be the number of reactions in pathway *P*; let *R*_pres _= {*r*_1_, ..., *r*_*k*_} be the set of reactions present in *P*. Let *n*(*r*_*i*_) be the number of pathways in which reaction *r*_*i *_appears. Then the "(all) normalized evidence information content" is defined as

This feature measures how strongly the evidence for pathway *P *is specific to *P*; reactions that are present in *P *and appear in few other pathways contribute greater weight. Normalizing by *m *downweights pathways with several promiscuous reactions, relative to pathways with unique reactions, but fewer of them present. Other variations omit the normalizing constant, or normalize by the number of reactions present.

Feature transformations were applied for use in some of the predictors. For the naïve Bayes predictors, numeric features were discretized using the following method. Each feature was discretized individually by considering each possible threshold between observed values of the feature in the training set. For each threshold, the information gain obtained by discretizing at that threshold is computed, and the threshold maximizing the information gain is selected. For the *k*-nearest neighbor predictors, feature values were standardized, subtracting from each feature value the mean value of that feature and dividing by its standard deviation.

### Performance Evaluation

For evaluating the performance of both individual features and prediction methods, we employed several widely used performance measures.

Performance of Boolean features or predictions is summarized by measures that depend on the number of true positive, false positive, true negative, and false negative predictions made. Let these numbers be *TP*, *FP*, *TN*, and *FN*. Also let *P *= *TP *+ *FN *be the total number of positives, *N *= *FP *+ *TN *the total number of negatives, and *G *= *P *+ *N *the total number of examples. We computed the following measures:

where *H *is the binary entropy function:(1)

used here as a measure of the purity of a collection of positive and negative examples.

For numeric features or predictions (in particular, predictions representing an estimated probability that a pathway is present), we compute the accuracy or F-measure obtained by optimizing over all possible thresholds of the feature or prediction value. The sensitivity and specificity are reported at the threshold that maximizes the accuracy, and the precision and recall are reported at the threshold that maximizes the F-measure. An additional performance measure specific to numeric predictions is the *area under the ROC curve *(AUC), where the *receiver operating characteristic (ROC) curve *plots sensitivity versus specificity over the entire range of prediction values.

To obtain unbiased estimates of the expected performance of our algorithms on new datasets, we do not report predictor performance on the entire gold standard dataset. Rather, we split the gold standard into training and test sets; we train each predictor (including feature selection, where appropriate) on the training set and compute its performance based on its predictions on the test set. The training/test split is done at random, with 50% of the examples selected for training and 50% held out for test. Each set of predictor performance results represents the average performance over 20 random training/test splits. The decision to use just 50% of the dataset for training was justified by learning curve experiments (see Additional file 2: Table S4, S5 and S6), in which we varied the fraction of examples used for training and examined performance on the held-out test data. We observed that performance increased rapidly up to approximately 1000 to 1500 examples, after which it increased only slightly.

### Training and Prediction

We evaluated four commonly used prediction algorithms: *naïve Bayes*, *k nearest neighbors*, *decision trees*, and *logistic regression*.

The *naïve Bayes *(NB) predictor is a simplified probabilistic model of the input features and the output to be predicted. The input features and output are represented as random variables, and the inputs are assumed to be conditionally independent given the output. Bayes' rule is used to compute the posterior probability that the pathway is present, given the observed input features. Let *Y *be a binary random variable indicating whether a given pathway is present; let *X*_1_, ..., *X*_*n *_be a collection of features with observed values *x*_1_, ..., *x*_*n*_. Then Bayes' rule gives us

where *P*(*Y *= 1) is the prior probability that a pathway is present. The prior probability is multiplied by the likelihood of the observed data, and divided by the normalization factor(2)

to obtain the posterior probability. In this work, we assume that all the observed features have been discretized to binary values. To obtain the full probability distribution *P*(*X*_1 _= *x*_1_, ..., *X*_*n *_= *x*_*n *_| *Y*) would require estimating an exponential number of parameters. The naïve Bayes model assumes that the feature variables *X*_*i *_are conditionally independent, given the output variable *Y*, so that posterior probability simplifies to

and(3)

Here, the number of parameters is linear in the number of features *n*. The parameters are estimated from the training data using maximum likelihood with pseudocounts.

The *k nearest neighbor *(*k*NN) predictor is an instance-based prediction method. The "training" phase simply involves recording the observed input and output data for the training instances. The predictor is parameterized by a positive integer *k *and a distance function *F*. To predict whether a given pathway is present, we select the *k *training instances that are most similar to the instance being classified; similarity is defined by applying the distance function *F *to the vectors of input feature values. Given the *k *nearest neighbors, a Boolean prediction is computed by majority vote of their output values. A numeric prediction can be computed as the fraction of the *k *nearest neighbors that are present.

We have omitted the performance results for *k*NN predictors, as initial experiments found *k*NN predictors to perform significantly worse than the naïve Bayes and decision tree predictors.

A *decision tree *(DT) predictor consists of a tree data structure where each internal node of the tree represents a test of one of the input features used for prediction, for example, testing whether the value of a Boolean feature is true, or whether the value of a numeric feature is less than a threshold value stored at the node. For each possible outcome of the test, there is a corresponding subtree. Each leaf node in the tree stores the numbers of present and absent training instances that satisfy all the tests between the root node and that leaf node. The decision tree prediction algorithm involves traversing the tree structure by applying the node tests to the instance being classified, starting with the test at the root of the tree, and continuing on to the subtree selected by the test. When a leaf node is reached, the counts of training examples at the leaf are used to make either a Boolean prediction (true if the majority of training instances at that node are present, false otherwise) or a numeric prediction (estimating the probability that the instance is present by the fraction of training instances at the node that are present).

We used the IND software package [12,13] for constructing decision trees and classifying instances using these trees. Several variations of the tree construction procedure were tried, including the use of different pruning techniques, and the use of Bayesian smoothing to obtain more accurate probability estimates at leaf nodes. In the Results, we report only the best-performing trees; for both single trees and bagged trees, these were the trees built using the Bayesian smoothed variant of the strict minimum message length (SMML) principle. The SMML training procedure aims to construct the tree for which the encoding cost of the tree plus the training data is minimized [14].

*Logistic regression *(LR) is a linear discriminative prediction method that models the logit of the output probability as a linear function of the features. Let *π *= *P*(*Y *= 1 | *X*_1 _= *x*_1_, ..., *X*_*n *_= *x*_*n*_). Then we assume that(4)

where *β *and **x **are vector forms of the parameters: (*β*_0_, *β*_1_, ..., *β*_*n*_) and feature values: (1, *x*_1_, ..., *x*_*n*_) (the extra 1 in the feature vector allowing for the intercept parameter *β*_0_). Solving for *π*, we have the predicted probability that a pathway is present as(5)

The maximum likelihood estimates of *β *are obtained using the iteratively reweighted least-squares (IRLS) algorithm ([15], Chapter 13).

#### Feature Selection

The set of 123 input features described in the section "Feature Extraction and Processing" includes many groups of features whose values are highly correlated with each other. To remove bias from the predictions, and to obtain a more computationally tractable set of features, it is necessary to perform feature selection to remove redundant features.

For decision tree predictors, feature selection is built into the tree construction methods, in the form of heuristics for deciding which feature to split on at each node, and which nodes to prune after a large tree has been built.

For naïve Bayes and logistic regression predictors, we used greedy hill-climbing (HC) search to perform forward selection against either of two information criteria: the Akaike (AIC) [16] or Bayes (BIC) [17]. Each of these criterion functions takes the form of a penalized log-likelihood, where models with greater numbers of parameters are penalized more heavily to avoid overfitting.

#### Ensemble Methods

In addition to using individual predictors, we investigated *ensemble methods *for prediction. These methods define procedures for training a collection of several different predictors; the prediction made by the ensemble is obtained by combining the predictions made by the members of the ensemble: either by taking a majority vote (to obtain a Boolean prediction) or by averaging (to obtain a numeric prediction). The particular ensemble methods used were *bagging *[18] and *random forests *[19].

In bagging (short for "bootstrap aggregating"), the training dataset is resampled (given the training dataset *D*, a new dataset *D'*, of the same size as *D*, is constructed by selecting instances from *D *at random with replacement) and a predictor is trained on the resampled dataset. This procedure (resample and train) is repeated *r *times, and the resulting set of *r *predictors is taken as an ensemble.

In more detail, for the naïve Bayes and logistic regression predictors, the resampled dataset was used to perform feature selection as described in the section "Feature Selection", and parameters for the resulting predictor were also computed from the resampled dataset.

The random forest method is an extension of bagging where, in addition to resampling the dataset, an additional element of randomness is introduced into the training procedure. For NB and LR models, we simply selected features at random. For decision trees we used the method described in [19], where rather than choosing the best of all features when splitting a node in the tree, we select the best feature from a small random subset of the features.

## Results

### Feature Performance

Tables 2, 3, and 4 show the highest-performing Boolean features, numeric features, and numeric features discretized as described in the section "Feature Extraction and Processing". The Boolean and discretized numeric features are ranked according to the information gain, and the numeric features according to the AUC; these measures are described in the section "Performance Evaluation". For comparison, Table 5 shows the performance of the existing PathoLogic algorithm.

**Table 2:** Best-performing Boolean features, ordered by information gain

Feature	ACC	SN	SP	FM	PR	RC	IG
has-enzymes	0.821	0.914	0.796	0.681	0.543	0.914	0.188
has-reactions-present	0.797	0.919	0.765	0.655	0.509	0.919	0.173
majority-of-reactions-present	0.872	0.707	0.916	0.699	0.69	0.707	0.165
some-initial-reactions-present	0.84	0.724	0.87	0.654	0.597	0.724	0.138
some-initial-and-final-reactions-present	0.864	0.605	0.933	0.651	0.706	0.605	0.136
mostly-absent-not-unique	0.215	0.163	0.229	0.08	0.053	0.163	0.133
all-initial-reactions-present	0.825	0.747	0.845	0.641	0.561	0.747	0.133
every-reaction-present	0.871	0.508	0.968	0.623	0.807	0.508	0.132
every-reaction-present-or-orphaned	0.871	0.508	0.968	0.623	0.807	0.508	0.132
taxonomic-range-includes-target	0.795	0.813	0.79	0.624	0.506	0.813	0.131

See section "Feature Extraction and Processing" and Section 1 of Additional file 2 for description of features.

Columns 2 through 8 correspond to various performance measures: ACC = accuracy; SN = sensitivity; SP = specificity; FM = F-measure; PR = precision; RC = recall; IG = information gain.

**Table 3:** Best-performing numeric features, ordered by AUC

Feature	AUC	max. ACC	SN (max. ACC)	SP (max. ACC)	max. FM	PR (max. FM)	RC (max. FM)
fraction-reactions-with-Enzymes	0.902	0.878	0.662	0.935	0.715	0.641	0.807
fraction-reactions-present	0.899	0.879	0.618	0.948	0.699	0.612	0.815
fraction-reactions-present-or-Orphaned	0.899	0.879	0.619	0.948	0.7	0.69	0.709
best-fraction-reactions-present-in-linear-path	0.898	0.879	0.662	0.936	0.703	0.682	0.726
evidence-info-content-norm-all	0.894	0.866	0.638	0.927	0.689	0.617	0.781
enzyme-info-content-norm	0.89	0.855	0.69	0.899	0.69	0.584	0.844
enzyme-info-content-unnorm	0.88	0.847	0.665	0.895	0.683	0.556	0.887
evidence-info-content-unnorm	0.875	0.841	0.526	0.925	0.657	0.511	0.918
num-reactions-with-enzymes	0.873	0.838	0.635	0.892	0.681	0.543	0.914
enzymes-per-reaction	0.871	0.842	0.686	0.883	0.688	0.567	0.875

See section "Feature Extraction and Processing" and Section 1 of Additional file 2 for description of features.

Columns 2 through 8 correspond to various performance measures: AUC = area under the ROC curve; max. ACC = maximum thresholded accuracy; SN (max. ACC) = sensitivity at maximum-accuracy threshold; SP = specificity at maximum-accuracy threshold; max. FM = maximum thresholded F-measure; PR (max. FM) = precision at maximum-F-measure threshold; RC (max. FM) = recall at maximum-F-measure threshold.

**Table 4:** Best-performing discretized numeric features, ordered by information gain

Feature	ACC	SN	SP	FM	PR	RC	IG
enzyme-info-content-norm	0.824	0.912	0.801	0.685	0.548	0.912	0.19
enzymes-per-reaction	0.822	0.914	0.798	0.683	0.545	0.914	0.189
fraction-reactions-with-enzymes	0.824	0.91	0.801	0.684	0.548	0.91	0.188
num-reactions-with-enzymes	0.821	0.914	0.796	0.681	0.543	0.914	0.188
num-enzymes	0.821	0.914	0.796	0.681	0.543	0.914	0.188
enzyme-info-content-unnorm	0.821	0.914	0.796	0.681	0.543	0.914	0.188
evidence-info-content-norm-all	0.821	0.893	0.802	0.677	0.545	0.893	0.179
best-fraction-reactions-present-in-linear-path	0.842	0.85	0.84	0.693	0.584	0.85	0.179
fraction-reactions-present	0.83	0.869	0.82	0.682	0.562	0.869	0.176
fraction-reactions-present-or-orphaned	0.852	0.817	0.861	0.698	0.609	0.817	0.176

See section "Feature Extraction and Processing" and Section 1 of Additional table 2 for description of features. See Table 2 for explanation of column headings.

**Table 5:** Performance of the existing, manually crafted PathoLogic algorithm for pathway prediction

ACC	SN	SP	FM	PR	RC	IG
0.91	0.793	0.94	0.786	0.779	0.793	0.233

See Table 2 for explanation of column headings.

### Predictor Performance

Table 6 shows the performance of several naïve Bayes predictors. For the predictors with random features, we first tested the effect of varying the number of features *r*, starting with *r *= 1, then ⌈1.1*r*⌉, below the total number of features, 123. We found that *r *= 37 optimized AUC, maximum accuracy, and maximum F-measure (averaging over three replicates for each value of *r*). For random "forests" of naïve Bayes predictors, we varied the number of components *c *starting from 1, then ⌈1.1*c*⌉, below 100 (an arbitrary cutoff), keeping the number of features in each component fixed at *r *= 37. We saw slight improvement in the performance measures up to approximately *c *= 60, which is reported in Table 6.

**Table 6:** Naïve Bayes performance

Predictor	AUC	max. ACC	SN (max. ACC)	SP (max. ACC)	max. FM	PR (max. FM)	RC (max. FM)
all features	0.91	0.883	0.763	0.915	0.736	0.68	0.804
random features (*r *= 37)	0.916	0.884	0.686	0.935	0.725	0.67	0.792
random forest (*r *= 37, *c *= 60)	0.924	0.888	0.709	0.936	0.737	0.693	0.791
HC-BIC feature selection	0.933	0.905	0.787	0.936	0.775	0.757	0.794
HC-AIC feature selection	0.938	0.905	0.78	0.938	0.777	0.759	0.796
bagged HC-BIC (*c *= 15)	0.945	0.908	0.751	0.949	0.782	0.761	0.805
bagged HC-AIC (*c *= 15)	0.946	0.909	0.757	0.949	0.78	0.767	0.796

See Table 3 for description of column headings. HC-BIC = hill-climbing on Bayes information criterion; HC-AIC = hill-climbing on Akaike information criterion.

For the bagged predictors with feature selection, we varied the number of components from 1 to 20, and found optimal performance over this range at approximately *c *= 15.

Table 7 shows the performance of several logistic regression predictors. For the predictors with random features, we again varied the number *r *of random features. We found rapid improvement in performance up to approximately *r *= 30, then slower improvement continuing to the largest value tested, *r *= 70. To limit the computational burden, we used *r *= 50 for the random features and random forest tests. For the random forest test, we used *c *= 8 components, obtained by testing *c *= 1 to 20. We tested bagged BIC predictors with *c *= 1 to 20 and found little variation in performance. We report the performance of bagged HC-BIC predictors for *c *= 8.

**Table 7:** Logistic regression performance

Predictor	AUC	max. ACC	SN (max. ACC)	SP (max. ACC)	max. FM	PR (max. FM)	RC (max. FM)
random features (*r *= 50)	0.939	0.902	0.732	0.947	0.768	0.74	0.8
random forest (*r *= 50, *c *= 8)	0.946	0.909	0.734	0.955	0.779	0.765	0.796
HC-BIC feature selection	0.948	0.91	0.738	0.956	0.785	0.765	0.808
HC-AIC feature selection	0.949	0.911	0.753	0.953	0.787	0.771	0.804
bagged HC-BIC (*c *= 8)	0.951	0.912	0.744	0.956	0.786	0.763	0.812

See Table 3 for description of column headings. HC-BIC = hill-climbing on Bayes information criterion; HC-AIC = hill-climbing on Akaike information criterion.

Table 8 shows the performance of decision tree-based predictors. For bagging, *c *= 25 trees were used. For random forests, *c *= 100 trees were used; at each step of growing the trees, *r *= 20 randomly selected features were tested as possible splits.

**Table 8:** Decision tree performance

Predictor	AUC	max. ACC	SN (max. ACC)	SP (max. ACC)	max. FM	PR (max. FM)	RC (max. FM)
single tree	0.946	0.909	0.714	0.961	0.777	0.755	0.802
bagged (*c *= 25)	0.953	0.911	0.729	0.961	0.787	0.77	0.808
random forest (*r *= 20, *c *= 100)	0.952	0.911	0.736	0.957	0.786	0.758	0.818

See Table 3 for description of column headings.

Table 9 shows the performance of predictors incorporating the PathoLogic prediction as a feature. The following models, performing comparably to PathoLogic in the results shown above, were tested: bagged naïve Bayes with HC-BIC feature selection; logistic regression with HC-AIC feature selection; and bagged decision trees. Allowing the PathoLogic prediction to be used as a feature does not uniformly improve the performance of these models, as can be seen in particular for the naïve Bayes model. However, bagged decision trees using the PathoLogic prediction feature dominate all other models in the AUC, maximum accuracy, and maximum F-measure, achieving a slight improvement over PathoLogic itself.

**Table 9:** Predictor performance using PathoLogic prediction as a feature

Predictor	AUC	max. ACC	SN (max. ACC)	SP (max. ACC)	max. FM	PR (max. FM)	RC (max. FM)
NB, bagged HC-BIC (*c *= 15)	0.936	0.912	0.775	0.948	0.79	0.779	0.801
LR, HC-AIC	0.949	0.913	0.756	0.954	0.789	0.773	0.806
DT, bagged (*c *= 25)	0.953	0.914	0.763	0.954	0.794	0.782	0.807

## Discussion

Our results demonstrate that machine learning methods perform as well as PathoLogic. Note that the results presented here do not show a full picture of the performance of the ML methods, which provide a tradeoff between sensitivity and specificity (precision and recall) by virtue of providing estimates of the probabilities of pathways being present in an organism, rather than simply binary present/absent calls. The performance of the ML methods can be slightly increased by using the PathoLogic prediction itself as an input feature.

**Figure 2:**
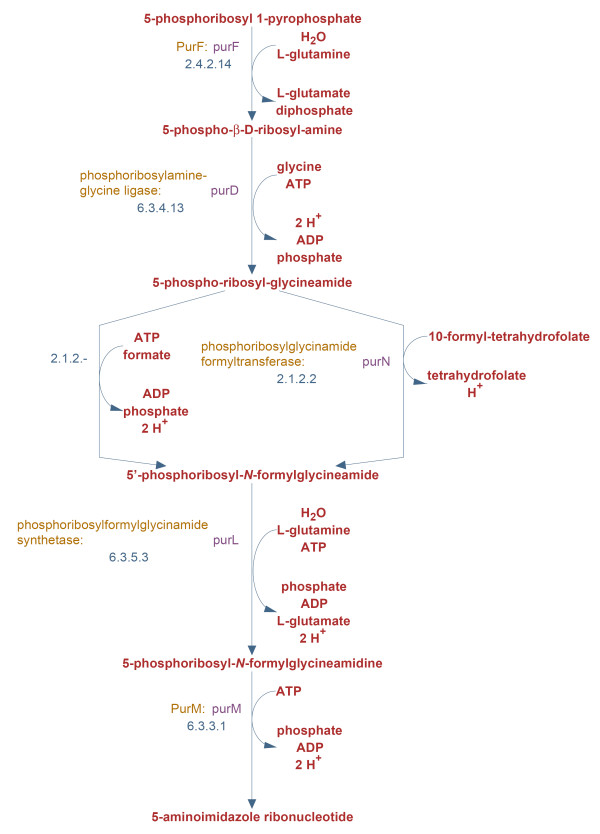
***Escherichia coli *K-12 MG1655 pathway 5-aminoimidazole ribonucleotide biosynthesis II**. This pathway is present in *E. coli*; PathoLogic excludes it while our machine learning methods consistently predict it to be present. See Table 10 for selected feature values for this pathway.

Figure 2 and Table 10 illustrate a key advantage of the ML methods over PathoLogic: the ability to dissect and display the evidence that led to a pathway being labeled as present or absent. Figure 2 shows the pathway diagram for *E. coli *pathway 5-aminoimidazole ribonucleotide biosynthesis II, including the enzymes present for five of the six reactions in the pathway. Table 10 shows the pathway's feature values for ten features selected (using the HC-AIC method) for inclusion in a naïve Bayes predictor trained on the entire gold standard. Also included in the table are the (base-2) log-odds ratios for the pathway's feature values in the trained predictor. These values summarize the evidence contributed by each feature for or against the pathway's presence. Positive scores represent evidence in favor of a pathway's presence, negative scores against. For example, a log-odds score of 1 for a particular feature would be obtained if that feature value were twice as likely to be seen for a present pathway as for a pathway that is absent (in the training dataset). In this case, the features enzyme-info-content-norm and taxonomic-range-includes-target-alt contribute fairly strong evidence for the pathway's presence, while a handful of the other features contribute weaker evidence either for or against the pathway's presence. A previous study [3] included a small-scale evaluation of the PathoLogic algorithm on *Helicobacter pylori*. The current work represents a major step forward in evaluating pathway prediction algorithms because we use a much larger gold standard dataset containing pathways from phylogenetically diverse organisms. To find ways to improve our algorithms, we examined incorrect pathway classifications made by either PathoLogic alone, by ML-based methods alone, or by both PathoLogic and the ML-based methods. We compared the predictions of PathoLogic to those of bagged decision trees on gold standard pathways from *E. coli *and *S. elongatus*.

**Table 10:** Feature values and log-odds ratios for a naïve Bayes predictor constructed with HC-AIC feature selection and trained on the entire gold standard, for pathway 5-aminoimidazole ribonucleotide biosynthesis II, shown in Figure 2.

Feature	Value	Log-odds
num-reactions	6	-0.04
enzyme-info-content-norm	0.47	2.19
is-subpathway	true	0.67
biosynthesis-pathway	true	0.22
majority-of-reactions-present-unique	false	-0.36
has-key-reactions	false	-0.07
some-key-reactions-are-present-alt	true	0.04
all-key-reactions-are-present-alt	true	0.04
taxonomic-range-includes-target-alt	true	1.71
subset-has-same-evidence	true	-0.44

The main cause of false negative classifications (not predicting to be present pathways that do occur in the organism) was inability of the enzyme matcher component of Pathway Tools (which is shared by PathoLogic and the ML methods) to find enzymes catalyzing some reactions in the pathway. In some cases, this failure can be attributed to incompleteness in the genome annotation. For example, many enzymes lack EC number annotations. Other enzymes have product names referring to several reactions; our enzyme name matcher currently ignores these names. Even for *E. coli*, where the annotation in EcoCyc is of high quality, our enzyme matching software cannot recover all enzyme/reaction matches, so that some evidence for pathway presence is missed. Issues with enzyme matching also contribute significantly to false positive predictions. GO function terms and EC numbers often refer to multiple MetaCyc reactions, which may participate in different pathways. This inability to distinguish which of several reactions an enzyme catalyzes inflates the evidence for some pathways. These observations suggest that an important direction for further improvement of the pathway prediction methods described here lies in improving the accuracy of enzyme/reaction mapping. Directions for this work might include extending genome annotation inputs to include confidence scores from upstream annotation methods. Data from sequence similarity or profile HMM search could also be incorporated into the pathway prediction process, using methods similar to those used in the Pathway Hole Filler component of Pathway Tools [5].

Another factor contributing to prediction errors is the existence of promiscuous reactions. The problem of promiscuous reactions can be addressed in part by making use of features involving the taxonomic range of a pathway and its key reactions. Most pathways in MetaCyc are annotated with an expected taxonomic range; if pathways sharing reactions have disjoint taxonomic ranges, this can help distinguish which pathway should appear in a given organism. PathoLogic enforces taxonomic range constraints rather strictly, by pruning pathways that are outside their expected taxonomic range, except those in which all reactions are present. We found that taxonomic range features were selected very frequently by our predictors; in decision trees, these features typically appeared very close to the root of the tree. (see Additional file 2: Table S1, S2 and S3 for lists of the features selected most frequently by our predictors. Figure 1 in Additionalfile 2 shows an example decision tree.) However, it appears that our predictors do not penalize as strongly as PathoLogic pathways that are outside their taxonomic range.

Key reactions are those reactions in a pathway for which the lack of enzyme is considered a very strong indication that the pathway does not occur in the organism. Key reaction features are frequently selected by our predictors. However, their current effect on pathway predictions is limited, because only 103 pathways in MetaCyc are currently annotated with key reactions (and thus, our training algorithms will assign little weight to these features). This will improve as we curate more key reaction data into MetaCyc.

### Related Work

The SEED [20,21] projects subsystems (which include metabolic pathways) into genomes. An algorithm infers proposed subsystems, which are checked and refined by curators. The inference algorithm has not been published, nor has its accuracy been measured. A related research algorithm is described [22]. Reactome [23] performs prediction of metabolic pathways based on genome information, but we have not been able to find a description of their algorithm nor an evaluation of its accuracy. KEGG [24] projects "pathway maps" based on genome information. KEGG pathway maps encompass multiple metabolic pathways from multiple organisms [25]; therefore, KEGG faces the pathway map prediction problem rather than the pathway prediction problem. We have been unable to find a description of KEGG's algorithm for map prediction or an evaluation of its accuracy. Methods for constructing flux-balance models usually predict the metabolic reaction network, but do not predict metabolic pathways [26].

Large-scale metabolic reconstructions at the pathway level have been used to perform phylogenetic reconstruction [27] and to associate metabolic pathways with phenotypes [28,29]. These efforts have typically used simple rules or scores for assessing the presence or absence of pathways. Liao et al. [27] required all reactions in a pathway to have enzymes in order for the pathway to be considered present. Kastenmüller et al. [28,29] developed a score similar to the "information content" features used in our predictors, computing the fraction of reactions present in the pathway, weighted according to the uniqueness of the reaction. We expect that such analyses could be improved by using the probabilities of pathway presence computed by our methods.

Several groups have developed methods for the reactome prediction problem; these methods include IdentiCS [30], metaSHARK [31], and Pathway Analyst [32,33], which use various sequence analysis techniques to assign enzymes to the reactions they catalyze. Published descriptions of IdentiCS and metaSHARK do not discuss how enzyme/reaction mappings are use to judge the presence or absence of pathways in an organism. Pathway Analyst considers a pathway present if and only if at least one reaction in the pathway has an enzyme. While such a rule may be acceptable for small-scale predictions (as described [33], Pathway Analyst includes only 10 pathway models, each encompassing several organism-specific variations of a high-level function such as "Alanine and aspartate metabolism" or "Glycolysis/gluconeogenesis"), it will not have sufficient accuracy for building hundreds of PGDBs from a large reference pathway database such as MetaCyc.

A number of techniques for discovering (or designing) novel pathways have been proposed, including search-based methods (e.g., [34]) which identify plausible paths between given input and output metabolites. Other approaches include searching for frequently-occurring patterns of molecular functions in biological networks [35] or kernel-based methods for learning associations between enzymes catalyzing successive functions in metabolic pathways [36]. These methods provide useful resources for identifying novel pathways, which are targets for experimental validation and inclusion in curated pathway databases such as MetaCyc. In this respect, pathway discovery methods are a useful complement to the pathway prediction methods we have described here.

## Conclusions

We have demonstrated the application of machine learning methods to the problem of metabolic pathway prediction from an annotated genome. A key product of this work has been the development of a large "gold standard" pathway prediction dataset, which we have used to validate our methods. The development of the gold standard pathway dataset provides a important foundation for future work on pathway prediction, by our group and by others working on this important task.

Our results show that general machine learning methods, provided with a well-designed collection of input features, can equal the performance of an algorithm that has been developed and refined over approximately a decade. We observed that a small number of features carry most of the information about whether a pathway occurs in a target organism. In particular, the fraction of reactions in the pathway with enzymes was the single most informative numeric feature. Whether the curated taxonomic range of the pathway includes the target organism - a test introduced into PathoLogic since the previous published description [3] - is also highly informative. All of the ML predictors we evaluated tended to include these features. The predictors often also included other features - newly developed in this work - which are less informative individually, but can contribute to prediction performance in the context of an automatically trained ML predictor.

Moreover, the machine learning approach has several benefits over the older algorithm. The machine learning approach decomposes the problem into three essential steps: (1) procuring labeled training data; (2) developing a modular library of useful features; (3) applying a general prediction algorithm. (See Figure 1.) Each of these steps can be optimized in the future to yield continued improvements in pathway prediction performance. As more PGDB curation is performed, the set of available training data will grow.

New features can easily be implemented and tested in combination with existing features. New prediction algorithms can be implemented and tested. The machine learning algorithms we have applied do not simply call each pathway present or absent, but rather provide an estimate of the probability that a pathway is present. Thus, the resulting pathway predictions can be tuned by the user to suit different preferences for sensitivity versus specificity. Furthermore, the structure and parameters of the model are accessible, and can be used to explain predictions to users of the pathway prediction software.

## Authors' contributions

JMD, LP, and PDK designed the research. JMD and LP carried out the research. JMD, LP, and PDK wrote the manuscript. All authors have read and approved the final manuscript.

## Supplementary Material

Additional file 1 **Gold Standard Pathway Dataset.** A table containing the gold standard pathway dataset. Each entry is described by organism name, NCBI Taxonomy ID, pathway name, pathway ID in the MetaCyc database, and pathway status ("present" or "absent"). Tab-delimited text format. Click here for file

Additional file 2 Supplementary Material, including additional tables and figures. Click here for file
