# Supplementary material for: Machine learning methods for metabolic pathway prediction
Source: BMC Bioinformatics. 2010 Jan 8;11:15. doi: 10.1186/1471-2105-11-15 (PMC3146072; doi:10.1186/1471-2105-11-15)
Supplement: Additional file 2 — Supplementary Material, including additional tables and figures. [file 1471-2105-11-15-S2.PDF]

Machine learning methods for metabolic pathway prediction
Supplementary Material

Joseph M. Dale, Liviu Popescu and Peter D. Karp

November 6, 2009

1 Features Used in Machine Learning Predictors

Here we list and describe the 123 features developed for use in the machine learning-based predictors.

1.1 Features in group PATHWAY-PROPERTIES

has-orphan-reaction (boolean)
True if the pathway has an orphan reaction.
has-spontaneous-reaction (boolean)
True if the pathway has a spontaneous reaction.
energy-pathway (boolean)
True if the pathway is an energy pathway.
deg-or-detox-pathway (boolean)
True if the pathway is a degradation pathway or a detoxification pathway.
detoxification-pathway (boolean)
True if the pathway is a detoxification pathway.
degradation-pathway (boolean)
True if the pathway is a degradation pathway.
biosynthesis-pathway (boolean)
True if the pathway is a biosynthetic pathway.
is-subpathway (boolean)
True if the pathway belongs to any superpathways in MetaCyc.
is-variant (boolean)
True if the pathway is a variant pathway.
has-unique-reactions (boolean)
True if the pathway contains reactions unique to the pathway in the reference.
multiple-reaction-pathway (boolean)
True if the pathway has more than one reaction.
single-reaction-pathway (boolean)
True if the pathway has only one reaction.
num-final-reactions (numeric)
The number of reactions in the pathway that have no successors in the pathway.
num-initial-reactions (numeric)

The number of reactions in the pathway that have no predecessors in the pathway.
num-unique-reactions (numeric)
Number of reactions in the pathway unique to the pathway in the reference.
num-reactions (numeric)
Number of reactions in the pathway.

1.2 Features in group REACTION-EVIDENCE

energy-pathway-mostly-missing (boolean)
True if the pathway is an energy pathway missing the majority of its reactions.
mostly-present (boolean)
True if (a) the pathway is missing at most one reaction; AND (b) the majority of reactions in the pathway are present; AND (c) no variant of the pathway has more evidence; AND (d) no pathway that is a subset of the pathway has the same evidence.
has-unique-enzymes (boolean)
Tests whether some enzyme catalyzes reactions only in this pathway.
has-enzymes (boolean)
True if there are enzymes catalyzing reactions in this pathway.
deg-or-detox-pathway-all-final-reactions-present (boolean)
True if the pathway is a degradation or detoxification pathway in which all final reactions are present.
deg-or-detox-pathway-all-initial-reactions-present (boolean)
True if the pathway is a degradation or detoxification pathway in which all initial reactions are present.
biosynthesis-pathway-all-final-reactions-present (boolean)
True if the pathway is a biosynthesis pathway in which all final reactions are present.
biosynthesis-pathway-all-initial-reactions-present (boolean)
True if the pathway is a biosynthesis pathway in which all initial reactions are present.
some-initial-and-final-reactions-present (boolean)
True if some initial reactions and some final reactions in the pathway are present.
some-final-reactions-present (boolean)
True if some final reactions in the pathway are present.
some-initial-reactions-present (boolean)
True if some initial reactions in the pathway are present.
all-initial-and-final-reactions-present (boolean)
True if all initial reactions and all final reactions in the pathway are present.
all-final-reactions-present (boolean)
True if all final reactions in the pathway are present.
all-initial-reactions-present (boolean)
True if all initial reactions in the pathway are present.
mostly-absent-not-unique (boolean)
True if the pathway is mostly absent, and no unique reactions are present.
mostly-absent (boolean)
True if no reactions in the pathway are present in the target, or if one reaction is present and this is a minority of the reactions in the pathway.
one-reaction-present-but-in-minority (boolean)
True if exactly one reaction in the pathway is present in the target, and this is a minority of the reactions in the

pathway.
every-unique-reaction-present-or-orphaned (boolean)
True if every reaction unique to the pathway in the reference is present in the target or an orphan in the reference.
every-unique-reaction-present (boolean)
True if every reaction unique to the pathway in the reference is present in the target.
every-unique-reaction-has-enzyme (boolean)
True if every reaction unique to the pathway in the reference has an enzyme in the target.
every-reaction-present-or-orphaned (boolean)
True if every reaction in the pathway is present in the target or an orphan in the reference.
every-reaction-present (boolean)
True if every reaction in the pathway is present in the target.
every-reaction-has-enzyme (boolean)
True if every reaction in the pathway has an enzyme in the target.
majority-of-reactions-absent (boolean)
True if the majority of reactions in the pathway are not present in the target.
majority-of-unique-reactions-present (boolean)
True if the majority of reactions that are unique to the pathway in the reference are present in the target.
majority-of-reactions-present-unique (boolean)
True if the majority of reactions in the pathway are present in the target and unique to the pathway in the reference.
majority-of-reactions-present (boolean)
True if the majority of reactions in the pathway are present in the target.
missing-at-most-one-reaction (boolean)
True if at most one reaction in the pathway is neither present in the target or an orphan in the reference.
has-unique-reactions-present (boolean)
True if some reaction in the pathway is present in the target and unique to the pathway in the reference.
has-reactions-present (boolean)
True if some reaction in the pathway is present in the target.
enzyme-info-content-norm (numeric)
Enzyme information content of the pathway, normalized by the number of enzymes catalyzing reactions in the pathway.
enzyme-info-content-unnorm (numeric)
Total enzyme information content of the pathway (unnormalized).
fraction-unique-enzymes (numeric)
Fraction of enzymes catalyzing reactions in this pathway that catalyze reactions only in this pathway.
num-unique-enzymes (numeric)
Number of enzymes that catalyze reactions in this pathway, and in no other pathways.
enzymes-per-reaction (numeric)
Number of enzymes catalyzing reactions in this pathway, divided by number of reactions.
num-enzymes (numeric)
Number of enzymes catalyzing reactions in this pathway.
fraction-final-reactions-present (numeric)
Fraction of final reactions in the pathway that are present in the target.
fraction-initial-reactions-present (numeric)
Fraction of initial reactions in the pathway that are present in the target.
num-final-reactions-present (numeric)
Number of final reactions in the pathway that are present in the target.

num-initial-reactions-present (numeric)
Number of initial reactions in the pathway that are present in the target.
evidence-info-content-norm-present (numeric)
Evidence information content of the pathway, normalized by the number of reactions in the pathway that are present in the target.
evidence-info-content-norm-all (numeric)
Evidence information content of the pathway, normalized by the number of reactions in the pathway.
evidence-info-content-unnorm (numeric)
Total evidence information content of the pathway (unnormalized).
fraction-unique-reactions-present-or-orphaned (numeric)
Fraction of reactions unique to the pathway in the reference that are either present in the target or orphans in the reference.
fraction-reactions-present-or-orphaned-unique (numeric)
Fraction of reactions in the pathway that are unique to the pathway in the reference, and either present in the target or orphans in the reference.
fraction-reactions-present-or-orphaned (numeric)
Fraction of reactions in the pathway that are present in the target or orphans in the reference.
fraction-unique-reactions-present (numeric)
Fraction of reactions unique to the pathway in the reference that are present in the target.
fraction-reactions-present-unique (numeric)
Fraction of reactions in the pathway that are present in the target and unique to the pathway in the reference.
fraction-reactions-present (numeric)
Fraction of reactions present in the pathway.
fraction-reactions-unique-with-enzymes (numeric)
Fraction of reactions in the pathway that have enzymes in the target and are unique to the pathway in the reference.
fraction-unique-reactions-with-enzymes (numeric)
Fraction of reactions unique to the pathway in the reference that have enzymes in the target.
fraction-reactions-with-enzymes (numeric)
Fraction of reactions in the pathway that have enzymes in the target.
num-unique-reactions-present-or-orphaned (numeric)
Number of reactions in the pathway that are present in the target or orphans in the reference, and unique to the pathway in the reference.
num-reactions-present-or-orphaned (numeric)
Number of reactions in the pathway that are present in the target, or orphans in the reference.
num-unique-reactions-present (numeric)
Number of reactions in the pathway that are present in the target and are unique to the pathway in the reference.
num-reactions-present (numeric)
Number of reactions in the pathway that are present in the target.
num-unique-reactions-with-enzymes (numeric)
Number of reactions in the pathway that are catalyzed in the target and are unique to the pathway in the reference.
num-reactions-with-enzymes (numeric)
Number of reactions in the pathway that are catalyzed in the target.

1.3 Features in group DEAD-END-COMPOUNDS

has-dead-end-outputs (boolean)
True if some output compound of the pathway is a dead end.
has-dead-end-inputs (boolean)
True if some input compound of the pathway is a dead end.
has-dead-end-compounds (boolean)
True if some input or output compound of the pathway is a dead end.
dead-end-output-rarity (numeric)
Total rarity of dead end outputs of the pathway.
fraction-dead-end-outputs (numeric)
Fraction of output compounds of the pathway that are dead ends.
num-dead-end-outputs (numeric)
Number of output compounds of the pathway that are dead ends.
dead-end-input-rarity (numeric)
Total rarity of dead end inputs of the pathway.
fraction-dead-end-inputs (numeric)
Fraction of input compounds of the pathway that are dead ends.
num-dead-end-inputs (numeric)
Number of input compounds of the pathway that are dead ends.
dead-end-compound-rarity (numeric)
Total rarity of dead end compounds of the pathway.
fraction-dead-end-compounds (numeric)
Fraction of input or output compounds of the pathway that are dead ends.
num-dead-end-compounds (numeric)
Number of input or output compounds of the pathway that are dead ends.
num-input/output-compounds (numeric)
Number of (primary) input or output compounds of the pathway.
num-output-compounds (numeric)
Number of (primary) output compounds of the pathway.
num-input-compounds (numeric)
Number of (primary) input compounds of the pathway.

1.4 Features in group KEY-REACTIONS

all-key-reactions-are-present-alt (boolean)
True if the pathway has no key reactions, or all the key reactions of the pathway are present in the target.
all-key-reactions-are-present (boolean)
True if all the key reactions of the pathway are present in the target.
some-key-reactions-are-present-alt (boolean)
True if the pathway has no key reactions, or at least one of the key reactions of the pathway is present in the target.
some-key-reactions-are-present (boolean)
True if at least one of the key reactions of the pathway is present in the target.
has-key-reactions (boolean)
True if the pathway has key reactions curated in MetaCyc.
fraction-key-reactions-present (numeric)

Fraction of key reactions of the pathway that are present in the target.
num-key-reactions-present (numeric)
Number of key reactions of the pathway that are present in the target.

1.5 Features in group GENOME-CONTEXT

has-genes-in-directon (boolean)
True if there is some gene in the pathway such that the directon of the gene contains another gene in the pathway.
has-proximal-genes (boolean)
True if there is some gene in the pathway such that a window of 5 genes upstream and 5 genes downstream of the gene contains another gene in the pathway.
fraction-genes-in-directon (numeric)
Returns the fraction of genes in the pathway such that the directon of the gene contains another gene in the pathway.
num-genes-in-directon (numeric)
Number of genes in the pathway such that the directon of the gene contains another gene in the pathway.
fraction-proximal-genes (numeric)
Fraction of genes in the pathway such that a window of 5 genes upstream and 5 genes downstream of the gene contains another gene in the pathway.
num-proximal-genes (numeric)
Number of genes in the pathway such that a window of 5 genes upstream and 5 genes downstream of the gene contains another gene in the pathway.

1.6 Features in group PATHWAY-CONNECTIVITY

pathway-degree (numeric)
Number of pathways containing reactions present in the target, such that the reaction produces an input compound, or consumes an output compound, of this pathway.
pathway-output-degree (numeric)
Number of pathways containing reactions present in the target, such that the reaction consumes an output compound of this pathway.
pathway-input-degree (numeric)
Number of pathways containing reactions present in the target, such that the reaction produces an input compound of this pathway.

1.7 Features in group PATHWAY-HOLES

has-complete-path (boolean)
True if there is a complete path through the pathway from an input compound to an output compound passing only through reactions present in the target.
deg-or-detox-pathway-missing-final-steps (boolean)
True if the pathway is a degradation or detoxification pathway with a run of at least two holes at the end.
deg-or-detox-pathway-missing-initial-steps (boolean)
True if the pathway is a degradation or detoxification pathway with a run of at least two holes at the beginning.

biosynthesis-pathway-missing-initial-steps (boolean)
True if the pathway is a biosynthetic pathway with a run of at least two holes at the beginning.
biosynthesis-pathway-missing-final-steps (boolean)
True if the pathway is a biosynthetic pathway with a run of at least two holes at the end.
hole-run-in-middle (boolean)
True if there is a run of at least two pathway holes in the middle of the pathway.
hole-run-at-end (boolean)
True if there is a run of at least two pathway holes at the end of the pathway.
hole-run-at-beginning (boolean)
True if there is a run of at least two pathway holes at the beginning of the pathway.
best-fraction-reactions-present-in-linear-path (numeric)
The maximum, over all linear paths through the pathway from an input compound to an output compound, of the fraction of reactions in the path that are present in the target.
fragments-per-reaction-present (numeric)
Number of pathway fragments divided by number of reactions present in the pathway.
reactions-per-fragment (numeric)
Number of reactions in the pathway divided by number of pathway fragments.
fragments-per-reaction (numeric)
Number of pathway fragments divided by number of reactions in the pathway.
num-fragments (numeric)
Number of pathway fragments (groups of connected reactions present in the target, separated from other fragments by pathway holes).

1.8 Features in group OTHER-PATHWAY-EVIDENCE

subset-has-same-evidence (boolean)
True if some pathway that is a proper subset of the given pathway has the same evidence.
other-pathway-has-more-evidence (boolean)
True if the evidence for the pathway is a proper subset of the evidence for another pathway.
variant-has-more-evidence (boolean)
True if the evidence for the pathway is a proper subset of the evidence for a variant pathway.

1.9 Features in group TAXONOMIC-RANGE

taxonomic-range-includes-target-alt (boolean)
True if the taxonomic range of the pathway is empty or includes the target.
taxonomic-range-includes-target (boolean)
True if the taxonomic range of the pathway includes the target.

2 Feature Selection Frequency

We show which features were selected by naïve Bayes, logistic regression, and decision tree predictors, and the frequency with which each feature was selected. For each predictor, 100 trials were performed; in each trial, 50% of

Table 1: Features selected by HC-AIC feature selection for naïve Bayes predictors, ordered by frequency of selection. Features appearing with frequency less than 0.1 are omitted.

Feature	Frequency
taxonomic-range-includes-target-alt	0.99
all-key-reactions-are-present-alt	0.75
is-subpathway	0.71
enzyme-info-content-norm	0.63
biosynthesis-pathway	0.62
majority-of-reactions-present-unique	0.57
some-key-reactions-are-present-alt	0.41
energy-pathway-mostly-missing	0.34
all-key-reactions-are-present	0.34
subset-has-same-evidence	0.31
enzymes-per-reaction	0.28
pathway-input-degree	0.17
deg-or-detox-pathway-all-initial-reactions-present	0.17
deg-or-detox-pathway-all-final-reactions-present	0.16
num-reactions	0.12

the gold standard was selected at random; a predictor was built and the features appearing in the predictor were collected. The fraction of trials in which each feature was selected is reported. Features appearing with a frequency of less than 0.1 are omitted.

Table 1 shows the frequencies for HC-AIC feature selection on naïve Bayes predictors. Table 2 shows the frequencies for HC-BIC feature selection on logistic regression predictors. Table 3 shows the frequencies for SMML decision trees.

As another illustration of the features typically used by our machine learning predictors, Figure 1 shows a decision tree printed by the IND package. This tree was constructed on the entire gold standard dataset using the strict minimum message length (SMML) procedure as described in the main text.

3 ROC and Precision/Recall Graphs

We show example ROC curves and precision/recall curves for naïve Bayes, logistic regression, and bagged decision tree predictors, as well as the sensitivity/specificity and precision/recall points for PathoLogic.

Figure 2 shows the ROC curves, illustrating that bagged decision trees dominate the other methods, including PathoLogic. However, the margin over logistic regression is small.

Figure 3 shows the precision-recall curves. Again, bagged decision trees dominate the other predictors, with only a small margin over logistic regression. Near the precision/recall point achieved by PathoLogic, all predictors have similar performance.

4 Learning Curves

We show data from learning curve experiments for bagged decision trees and naïve Bayes predictors. Each experiment is the average of 10 replicates. For each replicate, the gold standard was split into 80% training and 20% test data. From the training data, we first selected 100 examples at random, then 500 examples including the first 100,

Table 2: Features selected by HC-BIC feature selection for logistic regression predictors, ordered by frequency of selection. Features appearing with frequency less than 0.1 are omitted.

taxonomic-range-includes-target-alt	1.0
enzyme-info-content-norm	0.87
fraction-reactions-with-enzymes	0.69
deg-or-detox-pathway-all-final-reactions-present	0.40
evidence-info-content-norm-present	0.37
has-enzymes	0.35
best-fraction-reactions-present-in-linear-path	0.31
has-genes-in-directon	0.29
num-initial-reactions	0.25
biosynthesis-pathway	0.23
all-key-reactions-are-present	0.21
is-subpathway	0.21
biosynthesis-pathway-all-initial-reactions-present	0.17
fraction-dead-end-inputs	0.16
has-unique-enzymes	0.16
taxonomic-range-includes-target	0.13
has-dead-end-inputs	0.11
num-input/output-compounds	0.10
all-key-reactions-are-present-alt	0.10

Table 3: Features used in SMML decision trees, ordered by frequency of selection. Features appearing with frequency less than 0.1 are omitted.

Feature	Frequency
taxonomic-range-includes-target-alt	1.0
taxonomic-range-includes-target	0.79
evidence-info-content-norm-all	0.67
fraction-reactions-with-enzymes	0.58
biosynthesis-pathway-all-final-reactions-present	0.42
enzyme-info-content-norm	0.35
has-enzymes	0.33
best-fraction-reactions-present-in-linear-path	0.31
has-unique-enzymes	0.25
biosynthesis-pathway-all-initial-reactions-present	0.23
missing-at-most-one-reaction	0.21
has-complete-path	0.19
majority-of-reactions-present	0.17
fraction-unique-enzymes	0.16
biosynthesis-pathway	0.16
enzymes-per-reaction	0.11
num-unique-reactions-with-enzymes	0.1
fraction-dead-end-inputs	0.1

```
enzyme-info-content-norm < 0.1322:
|   taxonomic-range-includes-target = nil:
|   |   fraction-final-reactions-present < 0.41667:
|   |   |   taxonomic-range-includes-target-alt = nil:  (0.9946 0.00537) absent
|   |   |   taxonomic-range-includes-target-alt = t:  (0.9562 0.04378) absent
|   |   fraction-final-reactions-present >= 0.41667:  (0.8758 0.1242) absent
|   taxonomic-range-includes-target = t:
|   |   has-enzymes = nil:
|   |   |   biosynthesis-pathway-all-final-reactions-present = nil:
|   |   |   |   num-final-reactions < 4.5:  0.7917 0.2083 absent
|   |   |   |   num-final-reactions >= 4.5:  0.0625 0.9375 present
|   |   |   biosynthesis-pathway-all-final-reactions-present = t:  (0.9653 0.03474) absent
|   |   has-enzymes = t:  0.4228 0.5772 t
enzyme-info-content-norm >= 0.1322:
|   taxonomic-range-includes-target-alt = nil:
|   |   best-fraction-reactions-present-in-linear-path < 0.96154:  (0.8648 0.1352) absent
|   |   best-fraction-reactions-present-in-linear-path >= 0.96154:  (0.5667 0.4333) absent
|   taxonomic-range-includes-target-alt = t:
|   |   fraction-reactions-with-enzymes < 0.69615:
|   |   |   has-unique-enzymes = nil:
|   |   |   |   fraction-dead-end-inputs < 0.83333:  (0.3586 0.6414) present
|   |   |   |   fraction-dead-end-inputs >= 0.83333:  (0.6314 0.3686) absent
|   |   |   has-unique-enzymes = t:  (0.1383 0.8617) present
|   |   fraction-reactions-with-enzymes >= 0.69615:  (0.08762 0.9124) present
```

Figure 1: An SMML decision tree constructed on the entire gold standard dataset, as printed by the IND package. Each line represents a node in the tree. Lines representing internal nodes show the test applied at that node to decide which of the node's children should be visited next when classifying an example. Lines representing leaf nodes show the test that is applied to reach that node, a probability distribution over the outcomes (*absent present*), and a hard classification for that node based on which of the *absent* or *present* probabilities is larger.

then 1000 examples including the first 500, and so on, up to 4000 examples. For each set of examples a predictor was trained on the selected examples and tested on the held-out test set.

Table 4 shows the results for bagged decision trees ($c = 20$ trees). Table 5 shows the results for naïve Bayes predictors with HC-AIC feature selection. Both tables show a rapid increase up to at most 2000 examples, with little improvement thereafter.

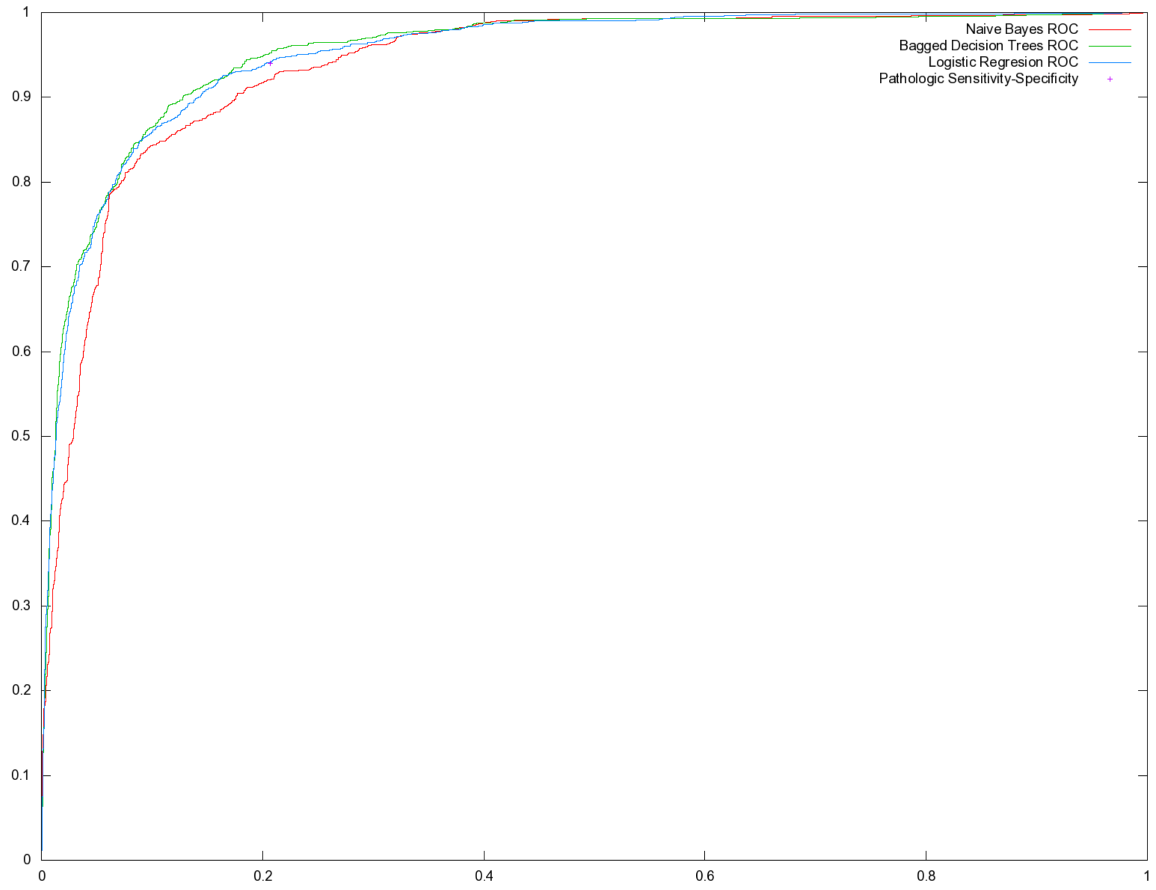

Figure 2: ROC curves.

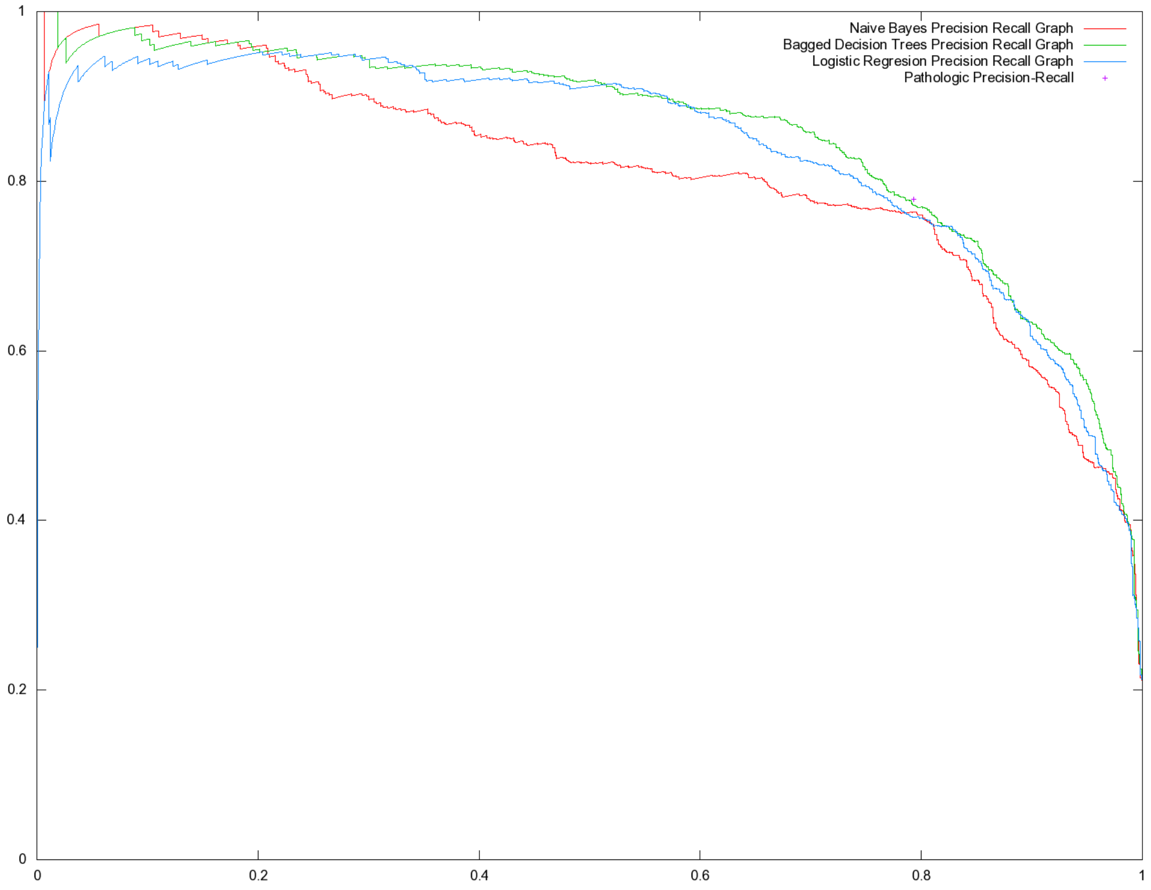

Figure 3: Precision/recall curves.

Table 4: Learning curve for bagged decision trees ($c = 20$). Size = number of examples in the training set. AUC = area under the ROC curve; max. ACC = maximum thresholded accuracy; SN (max. ACC) = sensitivity at maximum-accuracy threshold; SP = specificity at maximum-accuracy threshold; max. FM = maximum thresholded F-measure; PR (max. FM) = precision at maximum-F-measure threshold; RC (max. FM) = recall at maximum-F-measure threshold.

Size	AUC	max. ACC	SN (max. ACC)	SP (max. ACC)	max. FM	PR (max. FM)	RC (max. FM)
100	0.9252	0.8893	0.6931	0.9416	0.735	0.7044	0.7756
500	0.9434	0.9074	0.7449	0.9505	0.7769	0.7654	0.7939
1000	0.9455	0.9089	0.7175	0.9598	0.7783	0.7508	0.8121
1500	0.9488	0.9101	0.7241	0.9595	0.7806	0.7669	0.7989
2000	0.9497	0.9127	0.7377	0.9591	0.7839	0.7962	0.7756
2500	0.9505	0.9136	0.7142	0.9666	0.7848	0.8041	0.7712
3000	0.9503	0.9124	0.7223	0.9631	0.7831	0.776	0.792
3500	0.9508	0.9115	0.7235	0.9615	0.7829	0.7765	0.7925
4000	0.9515	0.9129	0.7342	0.9606	0.7857	0.7731	0.8013

Table 5: Learning curve for naïve Bayes predictors. See Table 4 for explanation of column headings.

Size	AUC	max. ACC	SN (max. ACC)	SP (max. ACC)	max. FM	PR (max. FM)	RC (max. FM)
100	0.9153	0.8993	0.7192	0.9472	0.7485	0.7639	0.7448
500	0.9348	0.908	0.7617	0.9466	0.7784	0.764	0.7986
1000	0.9366	0.9089	0.7789	0.9432	0.7806	0.7798	0.7821
1500	0.9339	0.9071	0.7641	0.945	0.7753	0.7677	0.7871
2000	0.9381	0.9109	0.788	0.9431	0.7865	0.7658	0.8104
2500	0.9364	0.9103	0.7823	0.9441	0.7849	0.7734	0.798
3000	0.9392	0.9108	0.7906	0.9424	0.7881	0.7682	0.8106
3500	0.9407	0.9116	0.8024	0.9401	0.7914	0.7687	0.8162
4000	0.9417	0.9103	0.8087	0.9371	0.7903	0.7676	0.8155

Table 6: Learning curve for logistic regression predictors. See Table 4 for explanation of column headings.

Size	AUC	max. ACC	SN (max. ACC)	SP (max. ACC)	max. FM	PR (max. FM)	RC (max. FM)
100.0	0.8611	0.8708	0.731	0.9071	0.7076	0.6687	0.7566
500.0	0.9387	0.9061	0.7276	0.9524	0.7618	0.8011	0.7276
1000.0	0.9421	0.9079	0.6929	0.9637	0.7621	0.7179	0.8126
1500.0	0.9426	0.907	0.705	0.9596	0.7718	0.7704	0.7739
2000.0	0.9444	0.9079	0.7277	0.9547	0.7711	0.7351	0.8113
2500.0	0.9457	0.9109	0.7352	0.9566	0.7774	0.7423	0.8188
3000.0	0.9458	0.9094	0.7175	0.9592	0.7713	0.7661	0.7767
3500.0	0.9445	0.9085	0.7092	0.9603	0.768	0.7507	0.7886
4000.0	0.9451	0.9082	0.7294	0.9547	0.7698	0.7671	0.7741
